# Dermatological adverse events in Chinese prostate cancer patients treated with the androgen receptor inhibitor apalutamide

**DOI:** 10.3389/fimmu.2025.1530919

**Published:** 2025-02-20

**Authors:** Qi Wang, Zhao-Ting Ren, Hui-Feng Wu, Hao-Chen Gu, Xia-Wei Li, Zhuang-Li Tang

**Affiliations:** ^1^ Department of Dermatology, The Second Affiliated Hospital, Zhejiang University School of Medicine, Hangzhou, China; ^2^ Department of Dermatology, Changxing People’s Hospital, Huzhou, China; ^3^ The Second Affiliated Hospital, Zhejiang University School of Medicine, Hangzhou, China; ^4^ Department of Urology, The Second Affiliated Hospital, Zhejiang University School of Medicine, Hangzhou, China; ^5^ Department of Gastrointestinal Surgery, The Second Affiliated Hospital, Zhejiang University School of Medicine, Hangzhou, China

**Keywords:** dAEs, dermatological adverse events, PSA, prostate-specific antigen, prostate cancer, apalutamide, Chinese population

## Abstract

**Background:**

Apalutamide, an androgen receptor inhibitor, has shown good efficacy in treating prostate cancer (PCa). However, dermatological adverse events (dAEs) are common and threatening, and relevant studies in China are limited.

**Methods:**

This was a retrospective, single-center analysis. We included PCa patients who were hospitalized and received apalutamide treatment at one comprehensive hospital in eastern China from August 2020 to March 2023. These patients were categorized into two groups for comparative analysis: those with (dAEs^+^) and without dAEs (dAEs^–^) based on the presence or absence of rash or itching following apalutamide administration. Demographics, PCa clinical and treatment data were extracted from the EMRS. The clinical features of dAEs were collected through follow-up calls.

**Results:**

Our study enrolled 90 individuals with an overall dAEs incidence of 50.0%. All dAEs occurred within one year following apalutamide use. Over half of dAEs^+^ patients suffered from pruritus, erythema or papules, and the dAEs were predominantly mild to moderate. Higher PSA levels were found in patients with dAEs.

**Conclusions:**

Apalutamide-associated dAEs are common in the Chinese population, among which the majority are mild to moderate, with pruritus, erythema, and papules ranking as the most prevalent symptoms. Elevated PSA levels were documented in patients with dAEs.

## 1 Introduction

Prostate cancer (PCa) is considered the second most common cancer and the fifth leading cause of cancer-related death among males globally, with an age-standardized incidence rate of 17.3/100000 in China (1, 2). Prostate-specific antigen (PSA) plays a pivotal role in PCa screening, with a probability of being greater than 50% for PCa if the PSA concentration exceeds 10 ng/mL (3). The Gleason score, the predominant grading system for histological assessment of PCa, is strongly associated with biological behavior and prognosis (4).

Apalutamide, an oral selective androgen receptor inhibitor that directly binds to the ligand-binding domain of the androgen receptor, is FDA-approved for treating patients with nonmetastatic castration-resistant prostate cancer or metastatic castration-sensitive prostate cancer. Despite its notable clinical efficacy, it can induce various adverse effects, with dermatological adverse events (dAEs) being particularly significant, with an estimated incidence of 23.4–27.1% (5–8). Notably, dAEs may endanger patients’ health and quality of life and lead to apalutamide dose interruptions. Although rare, severe dAEs associated with apalutamide, such as toxic epidermal necrolysis and Stevens-Johnson syndrome, have also been documented (9–11).

Interestingly, a low body mass index has been proposed to be a risk factor for such dAEs in the Japanese population, but little is known about this risk in other geographic regions (12, 13).

In terms of managing dAEs, various studies have suggested dose reductions or interruptions of apalutamide for severe dAEs (7, 14). Nevertheless, these approaches are largely empirical, and there is scarce guidance available specifically for dAEs associated with apalutamide.

Our study aimed to examine the incidence rate, clinical characteristics, and factors contributing to or alleviating apalutamide-associated dAEs in a Chinese population.

## 2 Materials and methods

This was a retrospective, single-center study. The ethics of the study were approved by the Second Affiliated Hospital, Zhejiang University School of Medicine (IRB 2023–0256), and verbal informed consent was obtained through telephone. The study was performed in accordance with the Declaration of Helsinki.

### 2.1 Patients

We retrospectively included treatment-naïve patients diagnosed with PCa and treated with apalutamide at the Department of Urology, Second Affiliated Hospital of Zhejiang University from August 2020 to March 2023, and followed them by telephone. For patients with moderate-severe dAEs diagnosed and treated by a dermatologist, we also referred to the visit records at that time. These patients were categorized into two groups for comparative analysis: those with dermatological adverse events (dAEs^+^) and those without (dAEs^–^). In our study, dAEs were defined as patient-reported skin lesions or discomfort, such as itching or skin dryness, following apalutamide administration.

### 2.2 Data collection

Demographics and PCa clinical data, such as PSA, testosterone, Gleason score, and treatment data, were extracted from the EMRS. The clinical features of dAEs, such as phenotype, time to onset, severity, and management, were collected through follow-up calls in March and April 2023 (**Supplementary Table S1**). According to the Patient-Reported Outcomes Version of the Common Terminology Criteria for Adverse Events (PRO-CTCAE) and Common Terminology Criteria for Adverse Events (CTCAE) Version 5.0, the severity of dAEs was classified as mild (Grade 1 in CTCAE/Mild in PRO-CTCAE), moderate (Grade 2 in CTCAE/Moderate in PRO-CTCAE) or severe (Grade 3 or higher in CTCAE/severe or higher in PRO-CTCAE) (15, 16). We mainly used the PRO-CTCAE for severity assessment and only rated the most severe disease out of multiple dAEs in individuals. For some of the dAEs that were not covered by the PRO-CTCAE, appeared as present/absent, or relied too much on subjective assessment in the PRO-CTCAE, to control for bias, we combined the body surface area score in the CTCAE for lesion area assessment. The specialists guided patients and their families through a rough estimation of the body surface area involved.

### 2.3 Statistical analysis

Owing to limitations associated with telephone follow-up, certain information was unavailable and thus excluded from the relevant statistical analysis. The normality of continuous data was assessed using the Shapiro-Wilk test. Normally distributed data are presented as the mean ± standard deviation and were analyzed with a t-test. Nonnormally distributed data are presented as medians with interquartile ranges (IQRs) and were analyzed with the Mann-Whitney U test, which was also used to compare unidirectionally ordered categorical variables such as the Gleason score. The Hodges–Lehmann estimate of the between-group difference with a 95% confidence interval was calculated with the corresponding P value from the Mann-Whitney U test. The chi-square test was used for the statistical analysis of dichotomous data. Statistical analysis was performed with SPSS 20.0, and graphs were created with GraphPad 8.0. A P value < 0.05 was considered to indicate statistical significance.

## 3 Results

### 3.1 Demographics

We retrospectively enrolled 99 patients. Unfortunately, 9 patients could not be reached for telephone follow-up or had communication difficulties, resulting in the inclusion of 90 patients (45 in the dAEs^–^ group and 45 in the dAEs^+^ group) in our study. The average age and body mass index (BMI) of patients in the dAEs^–^ group were 73.0 ± 9.0 years and 23.7 ± 3.2 kg/m^2^, respectively, whereas those of patients in the dAEs^+^ group were 73.6 ± 8.2 years and 23.2 ± 3.1 kg/m^2^, respectively. There were no significant intergroup differences regarding age (P = 0.732) or BMI (P = 0.443).

### 3.2 Clinical features and concomitant treatments of PCa

PSA in the dAEs^+^ group, with a median of 100.40 ng/mL, was far greater than that in the dAEs^–^ group (Hodges-Lehmann estimate of median difference, –43.09 ng/mL; 95% confidence interval [CI], –98.45–6.23; P = 0.012). Other parameters, including the serum testosterone level and Gleason score, were unremarkable (**Table 1**).

**Table 1:** Clinical characteristics of PCa patients in dAEs^–^ and dAEs^+^ groups.

	dAEs^–^ group (N = 45)	ND_dAEs_^–^	dAEs^+^ group (N = 45)	ND_dAEs_^+^	P value
PSA(ng/mL)	36.89 (IQR 17.16–95.66)	0	100.40 (IQR 27.72–299.63)	0	0.012*
Testosterone(nmol/L)	0.48 (IQR 0.27–0.72)	14	0.46 (IQR 0.25–0.79)	14	0.815
Gleason Score					
7	4 (8.9%)	3 (6.7%)	5 (11.1%)	4 (8.9%)	0.341
8	11 (24.4%)		16 (35.6%)		
9	22 (48.9%)		14 (31.1%)		
10	5 (11.1%)		6 (13.3%)		

dAEs^–^, patients without dermatological adverse events; dAEs^+^, patients with dermatological adverse events; IQR, interquartile range; N, number of patients; ND, no data; PCa, prostate cancer; PSA, prostate-specific antigen.

*P<0.05, statistically significant.

The concomitant treatments for PCa were similar across groups. Nearly 95% of the patients in each group received androgen deprivation therapy (P = 1.000). Abiraterone was only used in 9 (20.0%) dAEs^–^ patients and 5 (11.1%) dAEs^+^ patients (P = 0.245).

### 3.3 Clinical features of dAEs

**Figure 1:**
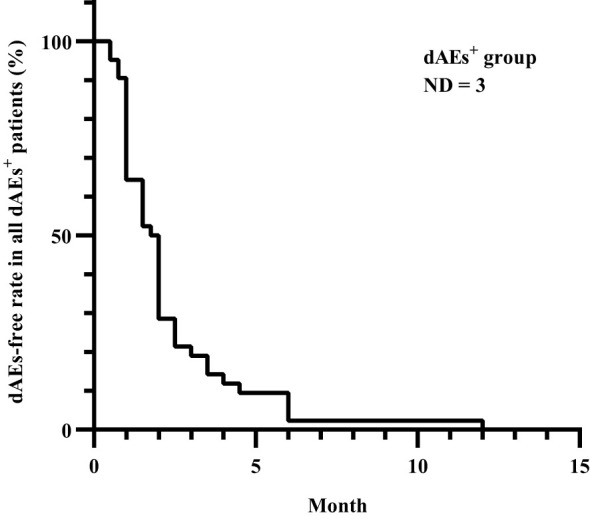
Time to dAEs onset after apalutamide use in all dAEs^+^ patients. All dAEs^+^ patients experienced dAEs within one year after apalutamide use, with a median time to onset of 1.9 months, except for 3 patients whose details could not be recalled. dAEs, dermatological adverse events; dAEs^+^, patients with dermatological adverse events; ND, no data.

All patients experienced dAEs within one year after apalutamide use, with a median time to onset of 1.9 months (**Figure 1**).

**Figure 2:**
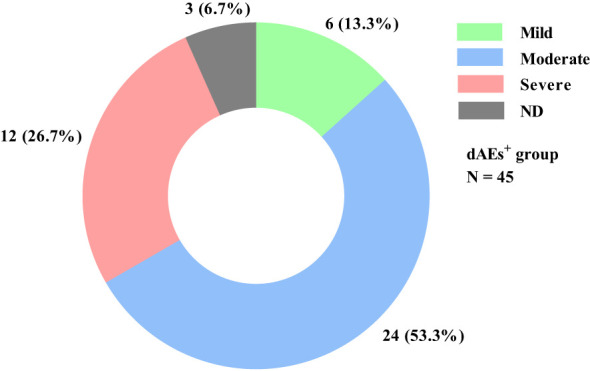
dAE severity distribution in all dAEs^+^ patients. Among the 45 dAEs^+^ patients, 6 (13.3%) and 24 (53.3%) patients experienced mild and moderate dAEs, respectively. Only 12 (26.7%) patients had severe dAEs, and 3 (6.7%) could not recall the details. dAEs, dermatological adverse events; dAEs^+^, patients with dermatological adverse events; N, the number of patients; ND, no data.

Pruritus, erythema, and papules were the most common, with incidences of 86.7%, 64.4%, and 53.3%, respectively (**Table 2**). Thirty (66.7%) patients had mild-moderate dAEs, whereas only 12 (26.7%) patients had severe dAEs (**Figure 2**). Among these 12 patients, pruritus, erythema and papules were also the most common. Hyperkeratosis and erosion/ulcer were relatively common, with incidences of 41.6% and 25.0%, respectively. There were no significant differences in dAE subtypes between mild-moderate and severe dAEs. Four of the 12 patients with severe dAEs, including 3 with Stevens–Johnson syndrome and one with the toxic epidermal syndrome, were definitively diagnosed at our institution.

**Table 2:** dAEs phenotypes in all dAEs^+^ patients (N = 45).

dAEs phenotypes	The number of patients
Pruritus	39 (86.7%)
Erythema	29 (64.4%)
Papule	24 (53.3%)
Xerosis	13 (28.9%)
Hyperkeratosis	11 (24.4%)
Erosion/Ulcer	4 (8.9%)

dAEs, dermatological adverse events; dAEs^+^, patients with dermatological adverse events; N, the number of patients.

### 3.4 Management after dAEs onset

After dAE occurrence, 25 (55.6%) patients maintained their previous apalutamide dose, 19 (42.2%) patients reduced their dose or frequency and withdrew or changed therapies, and one remaining patient could not recall the details. Systemic corticosteroids were prescribed to only 6 patients (13.3%). Twenty-eight (62.2%) dAEs^+^ patients achieved symptomatic relief, 12 (26.7%) patients had persistent dAEs, and 5 patients were unable to recall the details. Furthermore, it seems that dosage adjustment is not necessary, as no significant difference was observed among the different management practices (**Table 3**).

**Table 3:** Influence of apalutamide dose adjustments on dAEs improvement (N = 45).

	Dose adjustment	Dose maintenance	ND
Improvement	14 (31.1%)	14 (31.1%)	0 (0)
Non-improvement	4 (8.9%)	8 (17.8%)	0 (0)
ND	1 (2.2%)	3 (6.7%)	1 (2.2%)
P value	0.332		

dAEs, dermatological adverse events; N, number of patients; ND, no data.

However, it is not feasible to compare potential discrepancies in the course of dAE relief, as the majority of patients were unable to recall this information accurately.

## 4 Discussion

In 2020, PCa was the second most prevalent cancer and the fifth leading cause of male cancer fatalities worldwide (1). Apalutamide, a competitive androgen receptor inhibitor utilized in PCa treatment, exhibited strong efficacy in the phase 3 SPARTAN and TITAN trials. Nonetheless, dAEs are among the most common adverse reactions associated with their administration (5, 8).

Asians, especially Japanese individuals, seem to be at risk of experiencing dAEs linked to apalutamide, with reported incidence rates ranging from 38.7% to 56.0% (12, 13, 17–19). Additionally, in a phase 2 trial involving primarily a Chinese population, the occurrence rates of apalutamide-related dry skin and skin rash were 53.3% and 30.0%, respectively (20). Nonetheless, real-world data in this area remain scarce, particularly within China.

Unlike the incidence (28.4%) previously reported in a short-term retrospective analysis of the Chinese population (21), we found that as many as 50.0% of patients presented with dAEs regardless of severity, which is similar to data from Japanese cohorts. The long incubation period can be partially attributed to such variance.

The documented incubation period varies significantly in the literature, ranging from 51–74 days across different ethnic groups or demographic regions (7, 17, 22, 23). In our study, all dAEs emerged within one year following the initiation of apalutamide therapy, with a median onset time of 1.9 months, which is notably longer than that observed with traditional drugs. Nevertheless, Sasaki D et al. reported a median onset time of up to 6.9 months (12). Given these circumstances, arriving at a proper diagnosis may present challenges.

Apalutamide-associated dAEs include a range of phenotypes, with macular or maculopapular rash and xerosis being the most prevalent (7, 17, 22, 23). In our study, in addition to these two subcategories, increased attention was given to the elevated risk of pruritus, which can often be overlooked in clinical practice. Furthermore, there are numerous other manifestations, including stomatitis, erythema multiforme, urticaria, blistering, toxic epidermal necrolysis, lichenoid eruptions, erosions, psoriatic skin lesions, pustulosis, and drug reactions with eosinophilia and systemic symptoms syndrome (9, 11, 17, 23–31).

The outlook for patients with apalutamide-associated dAEs tends to be positive, with many individuals experiencing improvement without requiring systemic corticosteroids. In patients with moderate to severe dAEs, systemic corticosteroids, antihistamines, and emollients can provide relief. However, there remains debate over whether dose adjustment is necessary. Pan A et al. advised holding apalutamide until dAEs decreased to grade 1 or lower for severe cases of dAEs (7), whereas Oishi reported that dose reduction was not significantly linked to dAE incidence except in patients with smaller body sizes (18).

In our retrospective analysis, patients who received additional dosage adjustments or changes in frequency presented a comparable rate of improvement to those who remained on the previous dosage and frequency regimen. This can be attributed, at least in part, to the fact that 71.4–88.7% of apalutamide-associated dAEs are classified as grade 1/2 and rarely deteriorate (5, 7, 8, 17, 21, 22, 32). However, in several serious cases, i.e., toxic epidermal necrolysis and Stevens–Johnson syndrome, apalutamide can also be life-threatening (9–11). Therefore, whether and when physicians recommend drug adjustment should be thoroughly assessed.

The serum PSA level in the dAEs^+^ group was significantly greater than that in the dAEs^–^ group. Since few studies have reported the same conclusion to date, this finding needs to be confirmed by further studies.

BMI is regarded as a significant indicator of apalutamide-associated dAEs. A phase III study noted that apalutamide exposure tended to be greater in the Japanese population than in the non-Japanese population, attributing this observation to relatively low body weight (33). Sasaki D et al. and Katsuta M et al. reported that patients with small body sizes had notably greater rates of dAEs than those without small body sizes (12, 13). However, in our study, BMI did not appear to be associated with dAEs. Further research is warranted to uncover any potential underlying correlation.

Owing to the retrospective design of our study and the large variation in the time interval between dAEs onset and telephone follow-up, it is possible that some information provided by patients or their families during telephone follow-up may be inaccurate or uncertain. Additionally, patients with moderate-severe skin manifestations presented to dermatologists while the majority of patients had relatively mild dAEs that were recalled verbally over the telephone rather than being diagnosed by dermatologists, which may have resulted in information bias. In addition, sampling from a single center limits further generalization of our findings to a broader range. Additionally, the small sample size may introduce bias into the final conclusion. It is worth discussing whether the efficacy of apalutamide differs between patients with and without dAEs. However, due to the heterogeneity of patients’ tumor stage/grading, the diversity of antitumor treatments, and the relatively long follow-up period required for therapeutic effect assessment, obtaining robust information on the efficacy of apalutamide is expected to be difficult. Therefore, the efficacy of apalutamide was not included as one of our follow-up items. This issue is one of our limitations and deserves further research.

## 5 Conclusion

Apalutamide-associated dAEs remain quite common among the Chinese population, affecting 50% of dAEs^+^ patients irrespective of severity. All dAEs manifested within one year following apalutamide use, with a median onset time of 1.9 months. Pruritus, erythema, and papules emerged as the most prevalent symptoms, predominantly presenting as mild to moderate dAEs. The majority of patients with dAEs do not require additional systemic corticosteroids, and adjusting the apalutamide dosage seems to have little clinical significance. Elevated PSA levels were documented in patients with dAEs. However, no correlations were detected between other parameters, such as the Gleason score, BMI, age, or dAE occurrence.

## Data Availability

The raw data supporting the conclusions of this article will be made available by the authors, without undue reservation.

## Supplementary material

Supplementary Table 1 The list of follow up items through telephone.

## References

[1] Sung H Ferlay J Siegel RL Laversanne M Soerjomataram I Jemal A. Global cancer statistics 2020: GLOBOCAN estimates of incidence and mortality worldwide for 36 cancers in 185 countries. CA: Cancer J Clin. (2021) 71:209–49. doi: 10.3322/caac.21660 33538338

[2] Wang F Wang C Xia H Lin Y Zhang D Yin P. Burden of prostate cancer in China, 1990-2019: findings from the 2019 global burden of disease study. Front endocrinology. (2022) 13:853623. doi: 10.3389/fendo.2022.853623 PMC9175004 35692392

[3] Sekhoacha M Riet K Motloung P Gumenku L Adegoke A Mashele S. Prostate cancer review: genetics, diagnosis, treatment options, and alternative approaches. Molecules (Basel Switzerland). (2022) 27:5730. doi: 10.3390/molecules27175730 36080493 PMC9457814

[4] Humphrey PA. Histopathology of prostate cancer. Cold Spring Harbor Perspect Med. (2017) 7:a030411. doi: 10.1101/cshperspect.a030411 PMC5629988 28389514

[5] Smith MR Saad F Chowdhury S Oudard S Hadaschik BA Graff JN. Apalutamide treatment and metastasis-free survival in prostate cancer. New Engl J Med. (2018) 378:1408–18. doi: 10.1056/NEJMoa1715546 29420164

[6] Fang Z Xu Z Zhu W Yu M Ji C. A real-world disproportionality analysis of apalutamide: data mining of the FDA adverse event reporting system. Front Pharmacol. (2023) 14:1101861. doi: 10.3389/fphar.2023.1101861 37342589 PMC10277739

[7] Pan A Reingold RE Zhao JL Moy A Kraehenbuehl L Dranitsaris G. Dermatological adverse events in prostate cancer patients treated with the androgen receptor inhibitor apalutamide. J urology. (2022) 207:1010–9. doi: 10.1097/JU.0000000000002425 PMC9554897 35020444

[8] Chi KN Agarwal N Bjartell A Chung BH Pereira de Santana Gomes AJ Given R. Apalutamide for metastatic, castration-sensitive prostate cancer. New Engl J Med. (2019) 381:13–24. doi: 10.1056/NEJMoa1903307 31150574

[9] Guo ZJ Guo XJ Xu JF Hu FY Liang JZ Chen CX. Analysis for adverse events of apalutamide based on FAERS database. Chin J Pharmacoepidemiol. (2021) 30:744–9. doi: 10.19960/j.cnki.issn1005-0698.2021.11.006

[10] Liu F Cheng H Chen J Liu XJ. Case analysis on apalutamide-associated severe cutaneous adverse reactions. Eval Anal Drug-Use Hosp China. (2023) 23:756–60. doi: 10.14009/j.issn.1672-2124.2023.06.026

[11] Wang Q Cao H Zhang X Wu H Tang Z. Case report: Apalutamide-induced severe lethal cutaneous adverse effects in China. (2024) 14:1291564. doi: 10.3389/fimmu.2023.1291564 PMC10808516 38274795

[12] Sasaki D Hatakeyama S Tanaka T Okamoto T Yoneyama T Ohyama C. Impact of body size on skin-related adverse events in advanced prostate cancer treated with apalutamide: A multicenter retrospective study. Int J urology: Off J Japanese Urological Assoc. (2022) 29:772–3. doi: 10.1111/iju.14860 35285092

[13] Katsuta M Kimura T Tashiro K Murakami M Hata K Yanagisawa T. Low body weight as a risk factor for apalutamide-related cutaneous adverse events. Anticancer Res. (2022) 42:2023–8. doi: 10.21873/anticanres.15682 35347024

[14] Perez-Ruixo C Ackaert O Ouellet D Chien C Uemura H Olmos D. Efficacy and safety exposure-response relationships of apalutamide in patients with nonmetastatic castration-resistant prostate cancer. Clin Cancer research: an Off J Am Assoc Cancer Res. (2020) 26:4460–7. doi: 10.1158/1078-0432.CCR-20-1041 32561663

[15] NCI. National Cancer Institute Common Terminology Criteria for Adverse Events (CTCAE) v5.0 (2017). Available online at: https://ctep.cancer.gov/protocolDevelopment/electronic_applications/ctc.htm (Accessed March 10, 2023).

[16] Division of Cancer Control and Population Sciences NCI. Patient-Reported Outcomes version of the Common Terminology Criteria for Adverse Events (PRO-CTCAE™) (2024). Available online at: http://healthcaredelivery.cancer.gov/pro-ctcae/ (Accessed March 12, 2023).

[17] Uemura H Koroki Y Iwaki Y Imanaka K Kambara T Lopez-Gitlitz A. Skin rash following Administration of Apalutamide in Japanese patients with Advanced Prostate Cancer: an integrated analysis of the phase 3 SPARTAN and TITAN studies and a phase 1 open-label study. BMC urology. (2020) 20:139. doi: 10.1186/s12894-020-00689-0 32878613 PMC7465330

[18] Oishi T Hatakeyama S Tabata R Fujimori D Kawashima Y Tanaka R. Effects of apalutamide dose reduction on skin-related adverse events in patients with advanced prostate cancer: A multicenter retrospective study. Prostate. (2023) 83:198–203. doi: 10.1002/pros.24453 36314250

[19] Tohi Y Kato T Kobayashi K Daizumoto K Fukuhara H Ohira S. Real-world prostate-specific antigen response and progression to castration-resistant prostate cancer among men with metastatic castration-sensitive prostate cancer treated with apalutamide: a multi-institutional study in the Chu-shikoku Japan Urological Consortium. Jpn J Clin Oncol. (2024) 54:167–74. doi: 10.1093/jjco/hyad143 37840362

[20] Lee LS Sim AYL Ong CW Yang X Ng CCY Liu W. NEAR trial: A single-arm phase II trial of neoadjuvant apalutamide monotherapy and radical prostatectomy in intermediate- and high-risk prostate cancer. Prostate Cancer prostatic diseases. (2022) 25:741–8. doi: 10.1038/s41391-022-00496-8 PMC9705244 35091711

[21] Chen WQ Yuan RB Liu S Lai F Yang DY Li K. Apalutamide for the treatment of metastatic hormone-sensitive prostate cancer:a retrospective case series study. J Chongqing Med Univ. (2023) 48:949–52. doi: 10.13406/j.cnki.cyxb.003300

[22] Zhang YM Wen Y Xu P Zou JP Liu CX Chen BS. Treatment of skin rash induced by apalutamide in prostate cancer patients and literature review. J Mod Urol. (2022) 27:1009–12. doi: 10.3969/j.issn.1009-8291.2022.12.005

[23] Shima K Nomura T Yamada Y Usui S Kobayashi T Kabashima K. Maculopapular-type drug eruptions caused by apalutamide: case series and a review of the literature. J Eur Acad Dermatol Venereology: JEADV. (2022) 36:e113–e5. doi: 10.1111/jdv.17657 34510570

[24] Miyagawa F Akioka N Yoshida N Ogawa K Asada H. Psoriatic skin lesions after apalutamide treatment. Acta dermato-venereologica. (2022) 102:adv00659. doi: 10.2340/actadv.v102.858 35191508 PMC9631272

[25] Honda T Tohi Y Kaku Y Kimura N Kato T Haba R. Acute generalized exanthematous pustulosis during apalutamide treatment in a patient with prostate cancer. IJU Case Rep. (2022) 5:497–500. doi: 10.1002/iju5.12525 36341202 PMC9626358

[26] Flynn CR Liu SC Byrne B Ralph J Paz G Anwar S. Apalutamide-induced toxic epidermal necrolysis in a caucasian patient with metastatic castration-sensitive prostate cancer: A case report and review of the literature. Case Rep Oncol. (2023) 16:652–61. doi: 10.1159/000532009 PMC10601736 37900799

[27] Guglielmini PF Massone C Grasso C Francese A Vincenti M Chiodi S. A rare and severe lichenoid skin eruption after apalutamide treatment for prostate cancer. Annales dermatologie venereologie. (2023) 150:310–1. doi: 10.1016/j.annder.2023.09.001 37903707

[28] Cremante M Puglisi S Gandini A Guadagno A Catalano F Damassi A. Apalutamide-induced lichenoid reaction in a patient with non-metastatic castrate-resistant prostate cancer. J Oncol Pharm practice: Off Publ Int Soc Oncol Pharm Practitioners. (2023) 29:1748–53. doi: 10.1177/10781552231180598 37282554

[29] Hong YN Zhang YC Chen XF. A case of toxic epidermal necrolysis associated with apalutamide administration. Chin J Pharmacoepidemiol. (2023) 32:1188–93. doi: 10.19960/j.issn.1005-0698.202310013

[30] Huang LJ Lin S Li JY. One case of stevens-johnson syndrome caused by apalutamide. Pharm Clin Exp Res. (2023) 31:462–3. doi: 10.13664/j.cnki.pcr.2023.05.017

[31] Martin G Lambert E Wang GK. Drug reaction with eosinophilia and systemic symptoms (DRESS) syndrome caused by apalutamide: A case presentation. Cureus. (2023) 15:e41687. doi: 10.7759/cureus.41687 37575810 PMC10415966

[32] Ye JJ Bao YG Shen PF Liu ZH Yang L Zhang P. Clinical efficacy and safety of apalutamide in the treatment of prostate cancer: case series from West China Hospital. J Minimally Invasive Urol. (2022) 11:76–80. doi: 10.19558/j.cnki.10-1020/r.2022.02.002

[33] Uemura H Satoh T Tsumura H Arai G Imanaka K Shibayama K. Efficacy and safety of apalutamide in Japanese patients with nonmetastatic castration-resistant prostate cancer: a subgroup analysis of a randomized, double-blind, placebo-controlled, Phase-3 study. Prostate Int. (2020) 8:190–7. doi: 10.1016/j.prnil.2020.05.002 PMC7767934 33425798

