# Supplementary material for: Dermatological adverse events in Chinese prostate cancer patients treated with the androgen receptor inhibitor apalutamide
Source: Front Immunol. 2025 Feb 20;16:1530919. doi: 10.3389/fimmu.2025.1530919 (PMC11882852; doi:10.3389/fimmu.2025.1530919)
Supplement: Supplementary Table 1 — The list of follow up items through telephone. [file Table1.docx]

| Supplementary Table 1 The list of follow up items through telephone |
| --- |
| Question |
| Q1/Were there any skin lesions or discomfort like itching or skin dryness following apalutamide administration? (Yes/No) |
| Q2*/How long did the skin lesions or discomfort occur after apalutamide administration? |
| Q3*/Can you describe the exact manifestations of the skin lesions or discomfort? |
| Have you suffered from itching or pruritus? |
| Have you suffered from erythemas or red spots? |
| Have you suffered from papules? |
| Have you suffered from xerosis or skin dryness? |
| Have you suffered from desquamation, peeling or scaly rash? |
| Have you suffered from erosion, ulcer or blister? |
| In addition to the above manifestations, have you suffered from any other types of skin lesions or discomfort? |
| Q4*/what was the severity of your skin lesions or discomfort at its worst? (Mild/Moderate/Severe) |
| Q5*/Were you hospitalized for your skin lesions or discomfort at its worst? (Yes/No) |
| Q6*/If a palm-sized area is about 1% of your body surface area, what percentage of your body surface area was affected by your skin lesions or discomfort? |
| Q7*/Did you have any dose adjustments of apathetamine for your skin lesions or discomfort? (Yes/No) |
| Q8*/Did you use systemic corticosteroids for your skin lesions or discomfort? (Yes/No) |
| Q9*/Whether your skin lesions or discomfort improved or cured? (Yes/No) |
| List of abbreviations: Q, Question. |
| *Will be asked only when Q1 is Yes. |
